# The *Pseudomonas aeruginosa* Chemotaxis Methyltransferase CheR1 Impacts on Bacterial Surface Sampling

**DOI:** 10.1371/journal.pone.0018184

**Published:** 2011-03-22

**Authors:** Juliane Schmidt, Mathias Müsken, Tanja Becker, Zofia Magnowska, Daniela Bertinetti, Stefan Möller, Bastian Zimmermann, Friedrich W. Herberg, Lothar Jänsch, Susanne Häussler

**Affiliations:** 1 Department of Cell Biology, Helmholtz Center for Infection Research, Braunschweig, Germany; 2 TWINCORE, Center for Experimental and Clinical Infection Research, a Joint Venture of the Helmholtz Center for Infection Research and Medical School Hannover, Hannover, Germany; 3 Department of Biochemistry, University of Kassel, Kassel, Germany; 4 Biaffin GmbH & Co KG, Kassel, Germany; University of Osnabrueck, Germany

## Abstract

The characterization of factors contributing to the formation and development of surface-associated bacterial communities known as biofilms has become an area of intense interest since biofilms have a major impact on human health, the environment and industry. Various studies have demonstrated that motility, including swimming, swarming and twitching, seems to play an important role in the surface colonization and establishment of structured biofilms. Thereby, the impact of chemotaxis on biofilm formation has been less intensively studied. *Pseudomonas aeruginosa* has a very complex chemosensory system with two Che systems implicated in flagella-mediated motility. In this study, we demonstrate that the chemotaxis protein CheR1 is a methyltransferase that binds *S*-adenosylmethionine and transfers a methyl group from this methyl donor to the chemoreceptor PctA, an activity which can be stimulated by the attractant serine but not by glutamine. We furthermore demonstrate that CheR1 does not only play a role in flagella-mediated chemotaxis but that its activity is essential for the formation and maintenance of bacterial biofilm structures. We propose a model in which motility and chemotaxis impact on initial attachment processes, dispersion and reattachment and increase the efficiency and frequency of surface sampling in *P. aeruginosa*.

## Introduction

Biofilms are generally defined as sessile bacterial communities attached to a surface and embedded in a self-produced extracellular matrix. This polymeric matrix acts as a protective shield and - together with cellular appendages - facilitates adherence of the bacteria to each other and/or to surfaces. Biofilm communities represent not only the most prevalent bacterial mode of growth in the environment [1], but they have also become a focus of microbiological research due to their impact on industry and human health [2], [3]. It has been estimated that up to 80% of the bacterial infections in the industrialized countries are biofilm associated infections, refractory to antimicrobial therapy [4]. In order to develop novel strategies for biofilm control, it is critical to understand the adaptive pathways leading to the development and maintenance of bacterial biofilms.

So far, research on global gene or protein biofilm expression patterns did not succeed in the identification of a specific developmental biofilm program and thus putative novel targets for an anti-biofilm strategy. New knowledge about mechanisms involved in biofilm formation have recently been obtained by the use of optical tools to monitor *in vitro* grown biofilms. From those studies it has become evident that motility can have a profound impact on the colonization of surfaces [5]–[9]. However, the particular aspects of flagella and/or pili biogenesis and function, that are needed for biofilm formation in any species of bacteria, are not clearly defined. Pratt and Kolter have proposed a model for the initiation of *Escherichia coli* biofilm formation in which chemotaxis is dispensable but motility is required to overcome surface repulsion [9]. In contrast, there is evidence for the impact of chemotaxis on surface interactions and biofilm formation in other bacterial species such as *Aeromonas* spp. [10], *Vibrio cholerae* [11], *Pseudomonas aeruginosa* [12] and *Agrobacterium tumefaciens* [7].

Genome analysis reveals that a large number of environmental motile bacteria possess several genes involved in chemosensing and chemotatic signal transduction. Motile bacteria sense changes in the concentration of chemicals in their environment and respond in a behavioral manner [13]. The molecular mechanisms underlying bacterial chemotaxis have been studied extensively in the enteric bacteria *E. coli* and *Salmonella enterica* serovar Typhimurium [14], [15]. Chemotactic ligands are detected by cell surface chemoreceptors called methyl-accepting chemotaxis proteins (MCPs). Several homologous transmembrane receptors (MCPs) sense extracellular stimuli and produce signals that are transmitted to their cytoplasmic domains. These domains regulate an associated two-component phosphotransfer signal transduction system that controls flagellar rotation. The effect of ligand binding is counterbalanced by reversible MCP methylation providing the ability to detect chemical changes over time. Thereby, the opposing activities of two specific enzymes, CheR, a methyltransferase, and CheB, a methylesterase, control the MCP methylation level. CheR converts specific glutamic acid residues in the MCP cytoplasmic domain to glutamyl methyl esters, using *S*-adenosylmethionine (SAM) as the methyl donor [16]. CheB hydrolyzes those methylated residues, releasing methanol and restoring glutamic acid [17]. CheR activity is unregulated, whereas CheB is feedback-regulated by MCP output signals [18]. Studies of chemotaxis and MCP methylation in other organisms have revealed both, similarities and differences to the *E. coli*/*S.* Typhimurium chemotaxis pathway [19]–[24]. Given the different nature of the chemotactic system in the enteric bacteria and other environmental motile bacteria, it is not surprising that the roles chemotaxis plays in biofilm development are quite distinct.

*P. aeruginosa* inhabits a wide variety of environmental niches and is capable of locomotion by rotating a single polar flagellum. The bacterium has a very complex chemosensory system with more than 20 chemotaxis (*che*) genes in five distinct clusters and 26 *mcp*-like genes [25]. The Che and the Che2 systems, both homologous to the *E. coli* Che chemotaxis system, have been implicated in flagella-mediated chemotaxis [26]–[29], while genes in Pil-Chp cluster and Wsp cluster are involved in type IV pilus synthesis, twitching motility and biofilm formation, respectively [30]–[33]. Among the 26 MCPs of *P. aeruginosa*, nine have been identified as MCPs for amino acids, inorganic phosphate, oxygen, ethylene and volatile chlorinated aliphatic hydrocarbons [34], whereas 3 MCPs were demonstrated to be involved in biofilm formation and biosynthesis of type IV pilus [32], [35], [36].

In this study we demonstrate that *P. aeruginosa* CheR1 is a chemotaxis protein methyltransferase which uses *S*-adenosylmethionine as a methyl donor to methylate the MCP PctA in response to amino acids. Furthermore, CheR1 activity is shown to be essential for flagella-mediated chemotaxis and involved in bacterial surface sampling which impacts on the biofilm structures.

## Results

### CheR1 methylates the methyl-accepting chemotaxis protein PctA using *S*-adenosylmethionine as a methyl donor

The PA01 PA3348 gene product is predicted to be a probable chemotaxis protein methyltransferase (CheR1, www.pseudomonas.com, [37]). This function was proposed based on limited amino acid identity (31%) to the experimentally studied CheR gene product in *E. coli* and a conserved domain for *S*-adenosylmethionine (SAM) binding.

In *E. coli,* CheR methylation activity is strongly enhanced upon binding to a conserved pentapeptide sequence (NWETF) at the C-terminus of highly abundant MCPs [38], [39]. Homologous pentapeptide structures are present in *P. aeruginosa* only in 2 of the 26 MCPs (CttP and Aer2 [26]). The latter are however organized on the chromosome in the vicinity of the Che2 system, whose predicted gene products (including CheR2) exhibit an even higher overall sequence identity to orthologous *E. coli* chemotaxis proteins [26]. Thus, we speculated that CheR1 might methylate the MCPs PctA, PctB and PctC despite the lack of the pentapeptide sequence. Those MCPs have been shown to be involved in the detection of amino acids [40], [41].

**Figure 1:**
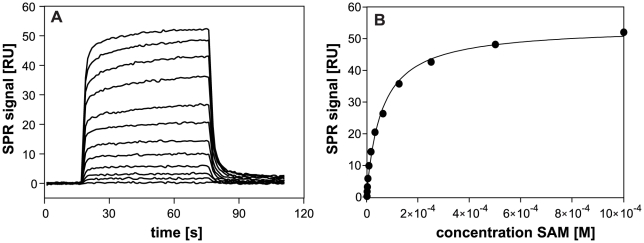
Surface plasmon resonance (SPR) analysis of the interaction of CheR1-His_6_ and SAM using a Biacore S51. CheR1 was immobilized on the sensor surface using standard amine coupling chemistry. The CheR1 interaction partner SAM was injected over the sensor surface. (A) SPR signals were detected for association (60 seconds) and dissociation (200 seconds) of SAM to CheR1. The graph shows the kinetic data for 12 SAM concentrations with data for the lowest (500 nM) to the highest (1 mM) SAM concentration displayed from bottom to top. (B) Data from (A) were analyzed with a steady state model. The resulting binding constant (*K*_D_) was determined to be 61 µM.

To characterize *P. aeruginosa* receptor methylation and to gain insight into methylation in MCPs lacking the pentapeptide motif, we performed *in vitro* methylation assays. We first expressed and purified *P. aeruginosa* His-tagged CheR1 protein and, since CheR uses *S*-adenosylmethionine (SAM) as a substrate for receptor methylation, we immobilized purified CheR1-His_6_ on a sensor chip and analyzed the interaction of CheR1 and SAM by the use of surface plasmon resonance. Figure 1 depicts the specific interaction of the ligand SAM and CheR1 thereby revealing a *K*_D_ value of 61 µM.

**Figure 2:**
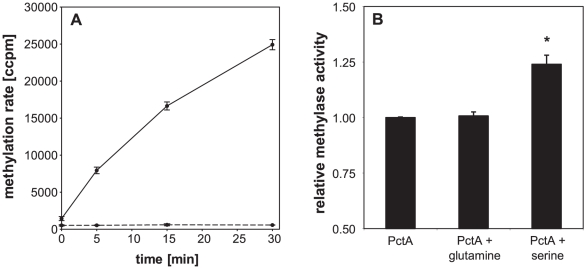
Methylation of the methyl-accepting chemotaxis protein PctA by CheR-His_6_ using [^3^H-methyl]-SAM as the methyl donor. (A) Methylation rate of *E. coli* HCB721 membranes containing no MCP (dashed line) or the *P. aeruginosa* MCP PctA (continuous line). (B) Methylation reactions carried out for 15 min in the presence of 1 mM serine resulted in an significantly increased methylation rate of the MCP PctA (* *p*<0.001, paired *t*-test), whereas the addition of glutamine had no effect. Representative data from one experiment out of at least two are shown. Error bars are standard deviations of three replica.

We next performed *in vitro* methylation assays by the utilization of purified *P. aeruginosa* His-tagged CheR1 protein, the methyl donor SAM and membranes from an *E. coli* strain (HCB721) effectively gutted of all the chemotaxis genes [42]. HCB721 cells were transformed with an IPTG inducible plasmid encoding the *P. aeruginosa* PctA receptor and membranes containing PctA were prepared as described under Materials and Methods. Figure 2A depicts the initial methylation rate of the PctA receptor and demonstrates that PctA was methylated by the methyltransferase CheR1.

Previous chemotactic assays of *pctA pctB pctC* triple mutants supplemented with either one of these MCP genes revealed that PctA, PctB and PctC detected 18 amino acids, 7 amino acids and 2 amino acids, respectively [41]. Among those amino acids, serine was detected by e.g. PctA whereas glutamine was only detected by PctB. We therefore tested the effect of those two amino acids on the methylation rate of PctA. In accordance to the previous chemotactic experiments, the presence of serine but not glutamine increased the methylation rate of PctA by at least 1.2-fold (p<0.001, Figure 2B).

### Motility defect of the methyltransferase *cheR1* mutant in *P. aeruginosa*

**Figure 3:**
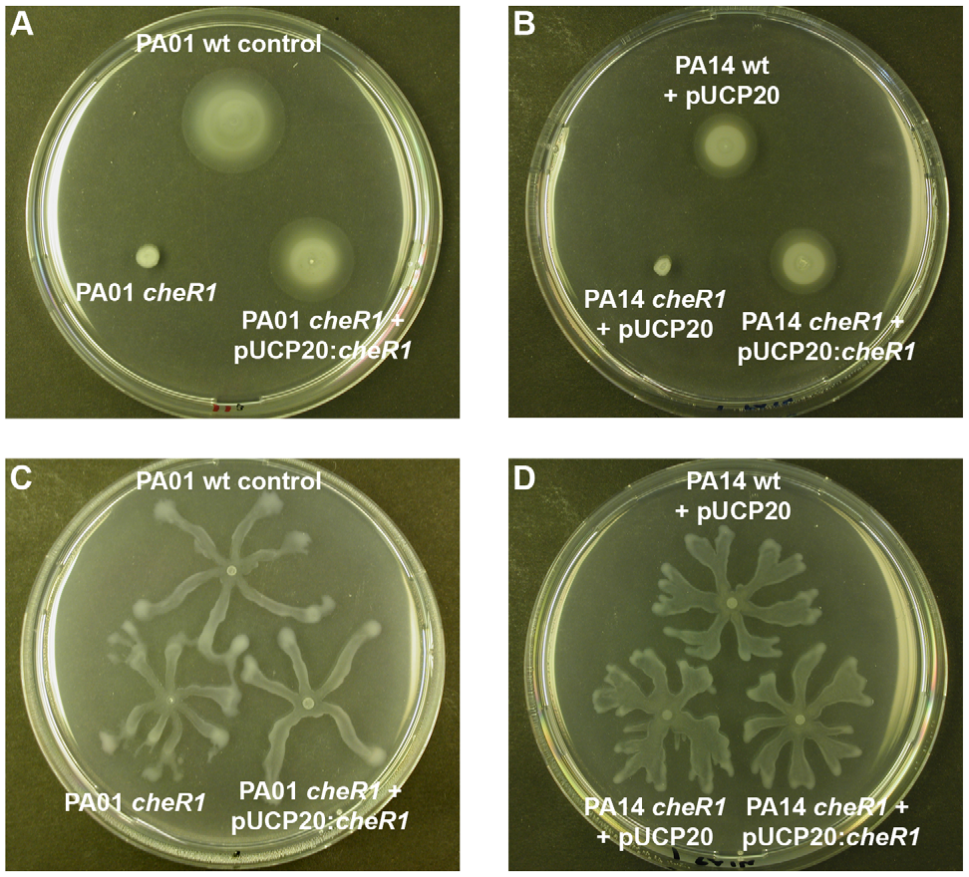
Motility phenotype of the *cheR1* mutant and the complemented strain of PA01 (A, C) and PA14 (B, D). (A, B) Swimming assays were performed on minimal medium soft agar plates supplement with 0.3% agar. The impaired swimming motility of the *cheR1* mutant can be complemented by providing the *cheR1* gene *in trans*. (C, D) Swarming assays were performed on minimal medium plates supplemented with 0.5% agar. All strains, the wild-type/wild-type control, the *cheR1* mutant and the complemented *cheR1* mutant, were capable of swarming.

In agreement with results described by Kato *et al.* [28], our analysis of *cheR1* transposon mutants in the PA01 and PA14 strain background (correct insertion of transposon was confirmed by PCR) revealed a severely impaired swimming motility in minimal medium soft agar plates (Figure 3A and B). Both *cheR1* transposon mutants formed only small diameter swim rings in contrast to the diffuse large diameter swim rings observed in the wild-type control and the complemented mutant strains. This phenotype defect is characteristic for mutants that cannot respond to a chemical gradient generated upon nutrient consumption and was not due to a defect in the growth rate (data not shown). By contrast, the *cheR1* mutant and complemented mutant strain were positive in swarming (Figure 3C and D) and twitching (data not shown) in both, the PA14 and PA01 strain background.

**Figure 4:**
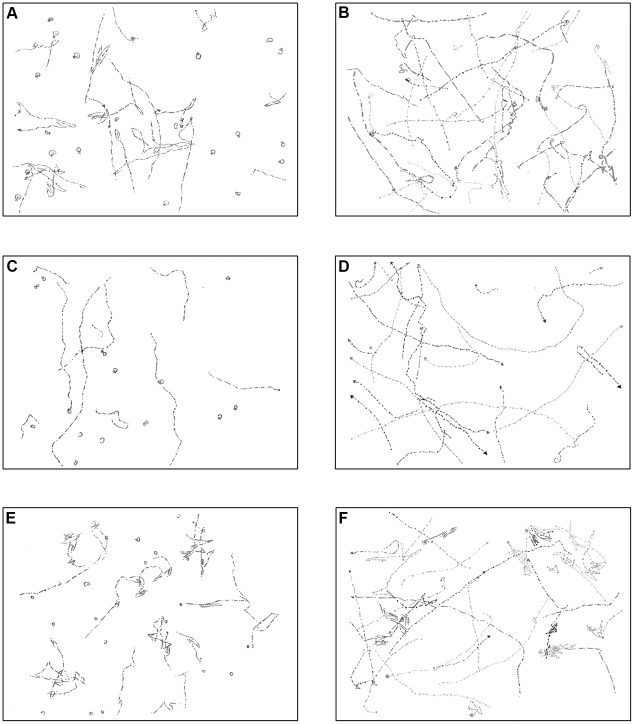
Motility defect of the *cheR1* mutant as observed by light microscopy. (A, B) The wild-type (A, PA01 wt control and B, PA14 wt) exhibits a straight forward - short backup/reversal - straight forward mode of swimming. (C, D) The *cheR1* mutant (C, PA01 and D, PA14) changes its direction less frequently than the wild-type and tends to swim straight. (E, F) The *cheR1* mutant complemented with pUCP20:*cheR1 in trans* (E, PA01 and F, PA14) is oscillating rapidly between forward and backward swimming.

In order to characterize the swimming motility defect in more detail we observed the cells by light microscopy. *E. coli* bacteria move unidirectional with periodic pauses that involve active tumbling, which reorients the cell prior to continued forward motion. This essential random movement can be biased through taxis mechanisms that modify the frequency of reorientations [14], [15]. Motility by other bacterial species including *P. aeruginosa* can differ significantly from the *E. coli* paradigm. As opposed to *E. coli* which possesses peritrichous flagella, *P. aeruginosa* possesses a single polar flagellum and swims in a straight, stop, turn mode (described by Pratt *et al.* [9]), driven by a (i) counter-clockwise rotation of the flagellum, (ii) brief reversal and (iii) reorientation. Both *P. aeruginosa* strains (PA01 and PA14) were motile and exhibited the run/stop/turn mode of swimming, although obviously the PA14 strain did not exhibit as much stops/reorientations as compared to the PA01 wild-type control under the tested conditions (Figure 4A and B). The *cheR1* mutants were also motile, but tented to swim straight and exhibited less frequent stops/reorientations as opposed to the PA01 and PA14 wild-type controls (Figure 4C and D). When the *cheR1* mutants were complemented with *cheR1 in trans*, the effect of CheR became more pronounced. The complemented *cheR1* mutants exhibited clearly more reversals as compared with the respective wild-type control and *cheR1* mutant in both strain backgrounds (Figure 4E and F).

### Compromised biofilm formation of the *cheR1* mutant

**Figure 5:**
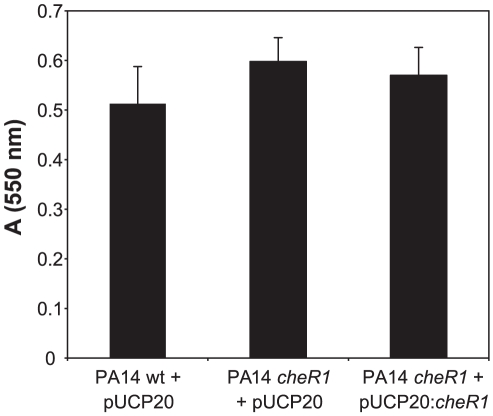
Crystal violet (CV) staining assay for the determination of attached biomass. PA14 wild-type carrying the vector control (pUCP20), the PA14 *cheR1* mutant carrying the vector control (pUCP20) and the complemented PA14 *cheR1* mutant (carrying pUCP20:*cheR1*) were grown in LB for 24 h at 37°C under static conditions before staining with CV. Representative data from one experiment out of five are shown. Error bars are standard deviations of eight replica.

The *cheR1* mutant of PA14 was further monitored for attachment and biofilm formation. We used the crystal violet (CV) staining assay for the determination of attached biomass following a 24 h incubation period of static cultures. As depicted in Figure 5, no clear difference in attached biomass could be observed between the PA14 wild-type, the PA14 *cheR1* transposon mutant and the complemented strain.

We next examined the formation of biofilms on the bottom of a 96-well plate. Confocal laser scanning microscopy of biofilms stained with the BacLight Live/Dead stain revealed a severe biofilm defect of the PA14 *cheR1* mutant after 72 h of growth in LB. A plasmid-borne copy of the deleted wild-type *cheR1* gene complemented the mutant phenotype (data not shown). To characterize the differences in biofilm formation in more detail, PA14 wild-type and *cheR1* mutant cells were tagged with plasmid-borne GFP and biofilm formation was monitored over time.

**Figure 6:**
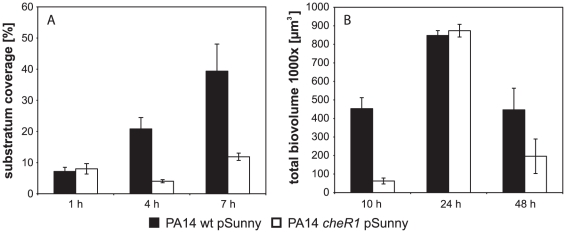
Differences in the development of GFP-expressing PA14 wild-type and *cheR1* mutant biofilms. (A) Quantification of the substratum coverage at the well-bottom of a 96-well plate after 1 h, 4 h and 7 h of growth in LB at 37°C. (B) Quantification of the biovolume after 10 h, 24 h and 48 h of growth in LB at 37°C.

After 1 h, only few cells were found at the bottom of a 96-well plate in both, the wild-type and the *cheR1* mutant, the majority of which was non-motile. In the following hours, the number of swimming bacteria increased. Thereby, bacteria of the wild-type strain accumulated at the bottom of the well but remained highly motile until the space was completely occupied by bacteria. By contrast, far less bacteria of the *cheR1* mutant strain were found in the proximity of the bottom. Surface sampling of motile bacteria proceeded only slowly. The difference in early substratum coverage by the wild-type and the *cheR1* mutant is displayed in Figure 6A and Figure S1.

**Figure 7:**
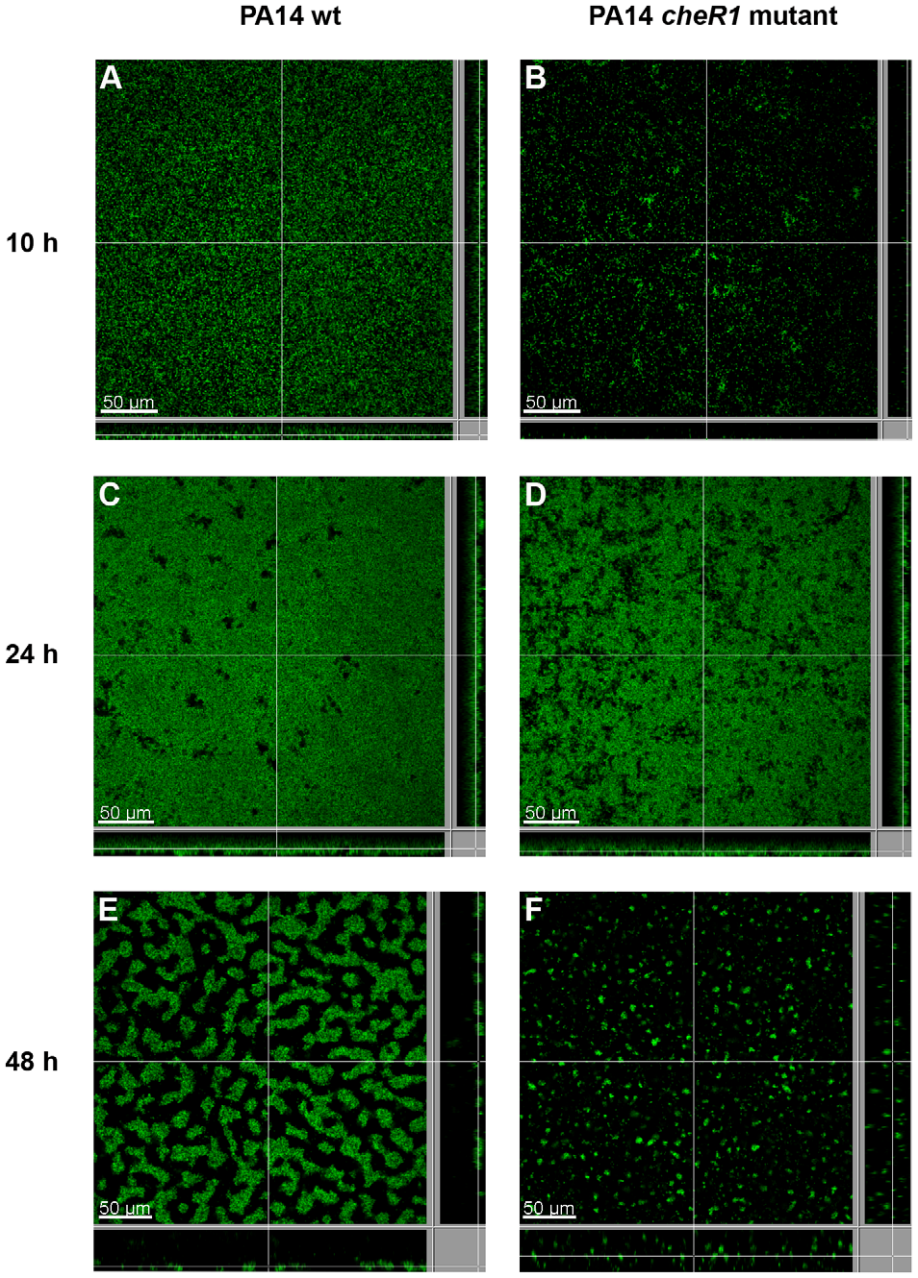
Confocal laser scanning micrographs of *P. aeruginosa* biofilms grown in LB. Biofilm development in a well of a 96-well plate at (A, B) 10 h, (C, D) 24 h and (E, F) 48 h is shown for (A, C, E) PA14 wild-type carrying the GFP-expression vector pSunny and (B, D, F) the PA14 *cheR1* transposon mutant carrying the GFP-expression vector pSunny. The central image shows a XY section through the biofilm and the flanking pictures show vertical sections. Scale bars, 50 µm.

We then monitored the formation of mature biofilms by three dimensional confocal laser scanning microscopy. After 10 h, biofilm formation of the *cheR1* mutant was severely delayed as compared with the wild-type (Figure 6B and Figure 7A and B). Nevertheless, after 24 h the structure and biovolume of *cheR1* biofilms were found to be very similar to that of wild-type biofilms (Figure 6B and Figure 7C and D). In the following, the wild-type and the *cheR1* mutant biofilm underwent structural rearrangement. Whereas the wild-type formed cohesive and densely packed biofilm structures, the mutant biofilm was characterized by a more loosely and in parts disconnected architecture and an overall lower biomass (Figure 6B and Figure 7E andF).

## Discussion

While signaling and chemotaxis have been most extensively explored in *E. coli* and *S.* Typhimurium, studies of other organisms revealed much more diversity and complexity in chemotactic signaling than had been previously anticipated. Most motile environmental bacteria have multiple homologues of the *E. coli che* genes and many more *mcp* genes than the five found in the enteric bacteria [15]. *P. aeruginosa* is chemotactic to most of the organic compounds that it can grow on and *P. aeruginosa* harbors as much as 26 genes that express homology to *E. coli mcp* genes [34]. *P. aeruginosa* has furthermore 5 clusters of *E. coli*-like chemotaxis genes. These include the *che* clusters cluster I (*cheY cheZ cheA cheB motC motD orf1 orf2 cheW*), cluster V (*cheV cheR*) and cluster II (*cttP cheY2 cheA2 cheW2 aer2 cheR2 cheD cheB2*), furthermore there is cluster III (*wspA wspB wspC wspD wspE wspF wspR*), as well as cluster IV (*pilG pilH pilI pilJ pilK chpA chpB chpC chpD chpE*). Whereas cluster I, V and II are evidently involved in flagella-mediated chemotaxis [26]–[29], cluster III is involved in the control of the expression of Pel and Psl exopolysaccharides [32] and cluster IV has been implicated in regulating twitching motility [31], [33].

Given the very different nature of the chemotactic systems present in the enteric bacteria versus environmental bacteria, it is not surprising that the roles the chemotaxis systems play are quite distinct. There are several reports concluding that - depending on the model system - chemotaxis is either required or dispensable for bacterial biofilm formation. *P. aeruginosa* harbors a complex chemotaxis system that seems to be involved in flagella-driven chemotaxis and in determining the cellular organization within biofilms. This renders *P. aeruginosa* probably a good model microorganism for investigating the roles of chemotaxis in bacterial adaptation to diverse environmental niches, including the establishment of structured bacterial communities.

In this study we provide evidence that CheR1 is a *P. aeruginosa* chemotaxis methyltransferase which transfers a methyl group from the methyl donor SAM to a methyl-accepting chemotaxis protein. As determined by surface plasmon resonance studies, CheR1 binds SAM at a *K*_D_ of approximately 60 µM, which is in the same order of magnitude as has been demonstrated for *Salmonella* CheR [43]. It has previously been shown that several amino acids are detected by the MCPs PctA, PctB and/or PctC in *P. aeruginosa* [41]. In accordance with these results, we could demonstrate in this study that *in vitro* methylation of the MCP PctA by CheR1 could be enhanced by the addition of serine but not of glutamine, the latter of which was shown to be specifically detected by PctB [41]. In *E. coli,* highly abundant transmembrane receptors harbor a conserved NWETF motif at the extreme C-terminal end of the MCP which recruits CheR to the receptor cluster and is required for efficient methylation and demethylation [38], [39]. Here we demonstrate that the MCP PctA was methylated by CheR1 despite the lack of a conserved C-terminal pentapeptide sequence. Similarly, a pentapeptide-independent methyltransferase has been characterized in the thermophilic bacterium *Thermotoga maritima* [44]. Co-crystallization of CheR from *S.* Typhimurium with the conserved NWETF pentapeptide demonstrated that the CheR β-subdomain is the region that interacts with the pentapeptide sequence [45]. β-subdomains can be divided into two groups: those with longer β-loops, found in CheR proteins from organisms containing MCPs with the pentapeptide recognition motif, and those with shorter β-loops, found in organisms that lack MCPs with the pentapeptide recognition motif [44]–[46]. Interestingly, in *P. aeruginosa*, CheR2 has a long β-loop and CheR1 has a short β-loop [44]. Moreover, only two out of 26 chemoreceptors have the conserved C-terminal pentapeptide motif and both genes encoding those chemoreceptors are located in the close proximity of the *che2* system. Overall, this might indicate that CheR2 uses a pentapeptide-dependent methylation mechanism, whereas CheR1 uses a pentapeptide-independent methylation mechanism and that this is one way of preventing crosstalk between different chemotaxis systems in *P. aeruginosa*.

A recent publication on the influence of motility and chemotaxis in *A. tumefaciens* revealed an impact of chemotaxis on both, attachment and biofilm formation [7]. In this study, the *P. aeruginosa cheR1* mutant did not exhibit a phenotype in the CV assay. Nevertheless, soft-agar plate assays and confocal microscope analysis of static biofilms clearly demonstrate that CheR1 activity is not only essential for flagella-mediated chemotaxis but that it is also involved in the formation of structured biofilms. Interestingly, CheR1 activity seems to be important at two developmental steps within the process of biofilm formation. Firstly, bacterial movement in close proximity to the surface enables surface sampling prior to irreversible attachment which seems to be important in the initial steps of biofilm formation and which seems to be promoted by the chemotaxis system. Secondly, the formation and consolidation of a more structured community seems to involve flagella-mediated chemotaxis. As far as we are aware, the only study examining biofilm formation of a *P. aeruginosa che1* chemotaxis mutant (*cheY*) was performed by Barken *et al.* [12]. They demonstrated that not type IV pili-driven but flagellum-driven motility is involved in the formation of cap structures in biofilms grown in a flow chamber irrigated with glucose minimal medium. Besides the *cheY* and *fliM* mutants, also a *P. aeruginosa rhlA* mutant deficient in biosurfactant production displayed reduced cap formation [47]. Since swarming motility requires both, the presence of biosurfactants and flagellar activity, Barken *et al.* [12] suggested that flagellum-driven surface-associated motility (by means of swarming) rather than directed motility (swimming in response to the sensing of certain metabolites) is required for cap formation. In this study, we used different experimental settings (static growth conditions, nutrient-rich medium), nevertheless, we also demonstrated a role of flagellum-mediated motility in the formation of structured biofilms. However, since our *cheR1* mutant displayed normal swarming motility on 0.5% agar plates, we rather hypothesize that chemotaxis driven swimming motility is necessary for formation of mature biofilm structures. A role for a substrate gradient that directs the motile bacteria has been suggested before [48].

In conclusion, one reasonable model for the role of motility and chemotaxis in biofilm formation in *P. aeruginosa* is that the efficiency and frequency of surface sampling may be influenced through chemotactic processes. This is important for initial sampling of bacteria to the surface and as the biofilm matures, chemotactic cues may stimulate dispersion. The released motile bacteria in the planktonic phase have the potential to colonize new sites and promote lateral expansion of the biofilm or to reattach in a coordinated chemotaxis-driven way, thus fine-tuning the architecture of the biofilm structures.

## Materials and Methods

### Bacterial strains, plasmids and growth conditions

Bacterial strains, plasmids and primers used in this study are listed in Table 1. Of note, transposon mutant *P. aeruginosa* PA01 ID 8031 from the Washington Genome Center [49] with a transposon insertion in open reading frame (ORF) PA4684 was used as wild-type control (PA01 wt control). ORF PA4684 is most likely coding for a non-functional gene product due to a large gene deletion [50], [51]. *P. aeruginosa* and *E. coli* strains were routinely cultured at 37°C in Luria-Bertani (LB) broth unless otherwise indicated. When required for plasmid or transposon selection, 100 µg/ml ampicillin and 50 µg/ml kanamycin were used for *E. coli* and 400 µg/ml carbenicillin, 15 µg/ml gentamycin, 150 µg/ml streptomycin and 25 µg/ml tetracycline respectively for the selection in *P. aeruginosa*.

**Table 1:** Strains, plasmids and primers used in this study.

Strain, plasmid or primer	Relevant characteristics^a^	Source or reference
Strains		
*E. coli* DH5α	F^-^ endA1 glnV44 thi-1 recA1 relA1 gyrA96 deoR nupG Φ80d*lacZ*ΔM15 Δ (*lacZYA-argF*)U169, hsdR17(r_K_^−^ m_K_^+^), λ–	[60]
*E. coli* BL21 (DE3)	F^-^ ompT hsdS_B_(r_B_^−^m_B_^−^) gal dcm	Stratagene
*E. coli* S17-1	C600::RP-4 2-(Tc::Mu) (Kn::Tn7) *thi pro hsdR hsdM*+*recA*	[61]
*E. coli* HCB721	Δ (*tsr*)7021 *trg*::Tn*10* Δ (*cheA-cheY*)::XhoI(Tn*5*), Km^r^, Tc^r^	[42]
PA01 wt control	*PA4684* transposon mutant from the Washington Genome Center PA01 mutant library, ID 8031, Tc^r^	[49]
PA01 *cheR1*	*cheR1* transposon mutant from the Washington Genome Center PA01 mutant library, ID 47867, Tc^r^	[49]
PA14 wt	Wild-type	[62]
PA14 *cheR1*	*cheR1* transposon mutant from the NR PA14 transposon mutant library, ID 36949, Gm^r^	[62]
**Plasmids**		
pUCP20	Shuttle vector, Ap^r^/Cb^r^	[63]
pUCP20:*cheR1*	*cheR1* (promoter region and gene) cloned into *Bam*HI-*Xba*I in MCS, Ap^r^/Cb^r^	This study
pET21a(+)	Plasmid for overexpression of proteins with C-terminal His_6_-tag, Ap^r^	Novagen
pET21a(+):*cheR1*	*cheR1* gene without stop codon cloned into *Nde*I-*Hind*III in MCS, Ap^r^	This study
pSunny	GFP expressing plasmid, Km^r^, Sm^r^	[59]
pHSe5:*tsr*	Plasmid expressing the *E. coli* MCP Tsr, inducible with IPTG, Ap^r^	[52], [53]
pHSe5:*pctA*	Plasmid expressing the *P. aeruginosa* MCP PctA, inducible with IPTG, Ap^r^	This study
**Primers** b		
PA3348 fPr1	5′-AAAACATATGGTGTCGGCAGCTAATGCG	
PA3348 rPr2	5′-GATCAAGCTTCTTGGCCCGGTAGAT	
PA3348 fPr3	5′-GATCGGATCCTTGCATACTTCGTTGTCC	
PA3348 rPr3	5′-GATCTCTAGACTACTTGGCCCGGTAGATG	
pctA fPr2	5′-GATCGGATCCATGATCAAAAGTCTGAAGTTCAGC	
pctA rPr2	5′-GATCAAGCTTTCAGATCTTGAAGCTGTCCAC	

a Ap^r^, ampicillin resistant; Cb^r^, carbenicillin resistant; Gm^r^, gentamycin resistant; Km^r^, kanamycin resistant; Sm^r^, streptomycin resistant; Tc^r^, tetracycline resistant

b Engineered restrictions sites are underlined

PCR amplifications were performed with Pfu polymerase using PA01 genomic DNA as a template. For complementation studies, the *cheR1* gene including 210 bp of the upstream region was amplified using the primer pair PA3348 fPr3/rPr3 and cloned into the *Bam*HI-*Xba*I sites of pUCP20. For expression of CheR-His_6_, the *cheR1* gene was amplified without its stop-codon using primer pair PA3348 fPr1/rPr2 and cloned into the *Nde*I-*Hin*dIII sites of pET21a(+) thereby fusing the gene to a C-terminal His_6_-tag. Obtained constructs were confirmed by restriction analysis and sequencing before being introduced into *P. aeruginosa* by electroporation (pUCP20:*cheR1*) or into *E. coli* BL21(DE3) by transformation (pET21a(+):*cheR1*).

The IPTG inducible *E. coli* Tsr expression plasmid pHSe5 [52], [53], kindly provided by Robert M. Weis (University of Massachusetts, Amherst), was modified for expression of the *P. aeruginosa* MCP PctA. The *tsr* sequence was removed by digestion with *Bam*HI and *Hin*dIII yielding in a vector backbone (4.8 kb) and a *tsr* fragment (2.4 kb). The vector backbone was gel-purified and ligated to the PCR amplified and *Bam*HI/*Hin*dIII digested *pctA* gene. The resulting construct, pHSe5:*pctA*, was transformed into chemically competent *E. coli* HCB721 cells.

### Overexpression and purification of CheR1

*E. coli* BL21 cells (Stratagene) carrying pET21a(+):*cheR1* were grown in LB supplemented with 100 µg/ml ampicillin and expression was induced with 0.1 mM isopropyl 1-thio-β-D-galactopyranoside (IPTG) at an OD_600_ of 0.5 to 0.7. The culture was shaken for 16 h at 20°C before harvesting the cells by centrifugation. The bacteria were resuspended in lysis buffer (50 mM NaH_2_PO_4_, pH 8.0, 300 mM NaCl, 10 mM imidazole) containing 1 mM DTT, 1 mg/ml lysozyme, protease inhibitors (Complete mini, EDTA free, Roche) and Benzonase Nuclease (Novagen) and passed through a French pressure cell at 16,000 psi (SLM-Aminco). Unbroken cells were removed by centrifugation at 37,500×*g* at 4°C for 45 min and the supernatant was incubated with nickel-nitrilotriacetic acid agarose resin (Qiagen) for 1 h at 4°C. The resin was washed with lysis buffer and proteins were eluted with 50 mM NaH_2_PO_4_, pH 8.0, 300 mM NaCl and 250 mM imidazole. After SDS-PAGE analysis, fractions containing pure protein were pooled and dialyzed for 16 h at 4°C in 50 mM NaH_2_PO_4_, pH 8.0, 300 mM NaCl.

### Surface plasmon resonance analysis

Surface plasmon resonance (SPR) interaction analyses were performed in 10 mM Tris, 300 mM NaCl, pH 8.0 (buffer A) at 25°C using a Biacore S51 instrument (GE Healthcare, Biacore). For covalent coupling of CheR-His_6_, carboxymethylated sensor chip surfaces (Series S CM5) were activated with a 1∶1 ratio of 0.4 M EDC (*N*-ethyl-*N'*-(3-dimethylaminopropyl)carbodiimide) and 0.1 M NHS (*N*-hydroxysuccinimide) for 10 min and purified CheR-His_6_ (40 µg/ml, 10 mM HEPES, pH 7.0) was injected for 20 min at a flow rate of 5 µl/min. Deactivation of the surface was performed using 1 M ethanolamine-HCl (pH 8.5) for 8 min.

Interaction analyses were performed in buffer A containing 0.005% (vol/vol) surfactant P20 by injection of increasing concentrations (500 nM-1 mM) of *S*-adenosylmethionine (SAM) at a flow rate of 30 µl/min. Association and dissociation signals were monitored for 60 s and 200 s, respectively. After subtracting the reference spot signal, resulting binding signals were fitted. Data evaluation was performed using the Biacore S51 evaluation software version 1.2.1.

### Preparation of membranes containing PctA

*E. coli* strain HCB721 [42], kindly provided by Howard C. Berg (Harvard University), was used to prepare membranes enriched for the MCP PctA. HCB721 is deficient in all known *E. coli* MCPs and all cytoplasmic chemotaxis proteins except the CheZ phosphatase and thus ensures that the overexpressed PctA receptor does not undergo posttranslational modification. Cells were grown at 30°C in 500 ml LB supplemented with 100 µg/ml ampicillin. At OD_600_ 0.5–0.7, PctA expression was induced with 1 mM IPTG. After 3 h of induction, cells were harvested by centrifugation and resuspended in buffer containing 100 mM potassium acetate, 50 mM HEPES, pH 7.5, 5 mM magnesium acetate, 0.05% (vol/vol) β-mercaptoethanol, protease inhibitors (Complete mini, EDTA free, Roche) and Benzonase Nuclease (Novagen). Cells were lysed by adding 1 mg/ml lysozyme and by passage through a French Pressure cell at least twice. After removing unbroken cells by centrifugation for 20 min at 7,000×*g*, the supernatant was loaded on a sucrose step gradient (0.5 M, 1.5 M and 2 M) and centrifuged at 100,000×*g* for 1 h at 4°C. The second band was removed, diluted 5 times with water containing protease inhibitors (Complete mini, with EDTA, Roche) and centrifuged for 1 h at 100,000×*g* at 4°C. The pellet was resuspended in a small amount of storage buffer (50 mM NaH_2_PO_4_, 1 mM EDTA, 10% (vol/vol) glycerol), analyzed on Coomassie-stained SDS PAGE and the total amount of protein concentration was determined by scanning densitometry against the Low Molecular Weight Calibration Kit (GE Healthcare) as a reference. The membrane samples were adjusted to a final protein concentration of 2 mg protein/ml in storage buffer and stored as single-use aliquots at −70°C.

### Receptor methylation assays

*In vitro* methylation assays were performed as previously described with slight modifications [54], [55]. In brief, the methylation reaction (final volume 100 µl) was carried out at 30°C in 50 mM NaH_2_PO_4_, pH 8.0, 300 mM NaCl containing 50 µl receptor-enriched membranes, 0.1 µM CheR1-His_6_ and with or without 1 mM serine or glutamine. After a pre-incubation step of 5 min, the reaction was started by adding 0.625 µM [^3^H-methyl]-*S*-adenosylmethionine (specific activity 80 Ci/mmol, Amersham). At indicated time points, 10 µl aliquots were removed, spotted on filter paper (1 cm^2^, Rotilabo, Roth) and quenched with 10% (wt/vol) trichloroacetic acid (TCA). The filters were washed twice with 10% (wt/vol) TCA and once with ethanol, for 15 min with gentle shaking. Air-dried filters were transferred into a 24-well sample plate, covered with 1 ml scintillation fluid and incubated over night before radioactivity was quantified using a microplate liquid scintillation counter (1450 MicroBeta TriLux, Wallac).

### Motility assays

Swimming and swarming motility assays were performed as previously described [56]. In brief, swimming was evaluated on BM2 glucose plates containing 0.3% agar and swarming on modified BM2 glucose plates containing 0.5% agar supplemented with 0.1% Casamino acids. Plates were inoculated with 1 µl of a pre-culture with OD_600_>1.0 and incubated over night at 37°C. Twitching assays were performed on LB plates with 1% agar by stab inoculation of single colonies with a toothpick. After 24–48 h incubation at 37°C, the diameter of the twitching zone at the plastic-agar interface was measured.

### Motility tracking

Bacterial cells were grown to exponential phase (OD_600_∼1.0) in LB, diluted 1∶200–1∶1000 in 0.9% NaCl containing 3% Ficoll (Sigma) and transferred into a 96-well plate with a thin glass bottom. Cells were monitored with an inverted microscope (Axiovert 135TV, Zeiss), equipped with a 25×/0.80 oil objective and a CoolSnap HQ2 camera (Visitron Systems) and operated with the MetaMorph software (version 7.5.3, Molecular Devices Corporation). Phase-contrast images visualizing cells near the glass bottom were acquired for 30 s at 5 frames per second, imported as stacks into ImageJ 1.42, where trajectories of swimming bacteria were monitored.

### Biofilm and attachment assays

Crystal violet staining of adherent cells was adopted from O'Toole and Kolter [57] with the following modifications. Overnight LB cultures were diluted to an OD_600_ of 0.02 with fresh medium. The bacterial suspensions were inoculated in PVC 96-well plates (Becton Dickinson Labware) with 100 µl per well (8 replica per strain), sealed with an air-permeable BREATHseal cover foil (Greiner Bio-One) and grown under static conditions at 37°C in an incubator with humid atmosphere. After 24 h of incubation, planktonic cells were removed and wells were washed with water prior to staining with 0.1% (wt/vol) crystal violet for 30 min at room temperature. Wells were carefully washed with water to remove excess staining solution, air-dried and the retained crystal violet was solubilized in 95% ethanol for 30 min at room temperature. For quantification, 125 µl of the resulting solution were transferred into a 96-well polystyrene microtiter plate (Nunc) and absorbance was measured at 550 nm.

Analysis of static biofilms grown at the bottom of 96-well plates was performed as previously described [58]. In brief, overnight LB cultures were adjusted to an OD_600_ of 0.02 with fresh medium and transferred into a half-area 96-well µClear plate (Greiner Bio-One, 100 µl/well, four replica per strain). The plate was covered with an air-permeable foil and incubated at 37°C in an incubator with humid atmosphere. After 24 h, bacteria were stained with 50 µl diluted staining solution (LIVE/DEAD BacLight Bacterial Viability kit, Molecular Probes/Invitrogen, final concentration of 1.4 µM Syto9 and 8.3 µM propidium iodide) and further incubated for 48 h at 37°C. Microscopic analysis of biofilm formation on the bottom of microtiter plates was performed using an Olympus Fluoview 1000 confocal laser scanning microscope equipped with a 40×/0.90 air objective. Image stacks were acquired in the center of each well with a step size of 2 µm. Images of each experiment were processed and analyzed as previously described [58] and visualized using the IMARIS software package (version 5.7.2, Bitplane).

To monitor biofilm formation in the microtiter plates over time, the constitutively GFP-expressing plasmid pSunny [59] was transferred into *P. aeruginosa* by conjugation and biofilms were grown, images acquired and data analyzed as described above except that the staining step was omitted. To determine the amount of bacteria attached to the substratum, we acquired single images at the bottom of the wells. We processed the images with a pseudo flat field filter to adjust uneven luminance. The filtered, gray-scale images were thresholded to segment objects' pixel area from background pixels. The percentage of attached bacteria was calculated by determining the ratio of object pixels to the total number of pixels. All processing steps were performed using ImageJ (version 1.43).

## Supporting Information

Figure S1 **Substratum coverage by GFP-tagged bacteria as monitored by CLSM.** The coverage of the well-bottom of a 96-well plate was monitored after (A, B) 1 h, (C, D) 4 h and (E, F) 7 h of growth in LB at 37°C. The cell clusters observed in (A) and (B) are likely to originate from cell clumps of over night grown pre-cultures used for inoculation. (A, C, E) PA14 wild-type and (B, D, F) PA14 *cheR1* transposon mutant.
(TIF)
