# Supplementary figures and images for: The Pseudomonas aeruginosa Chemotaxis Methyltransferase CheR1 Impacts on Bacterial Surface Sampling
Source: PLoS One. 2011 Mar 22;6(3):e18184. doi: 10.1371/journal.pone.0018184 (PMC3062574; doi:10.1371/journal.pone.0018184)

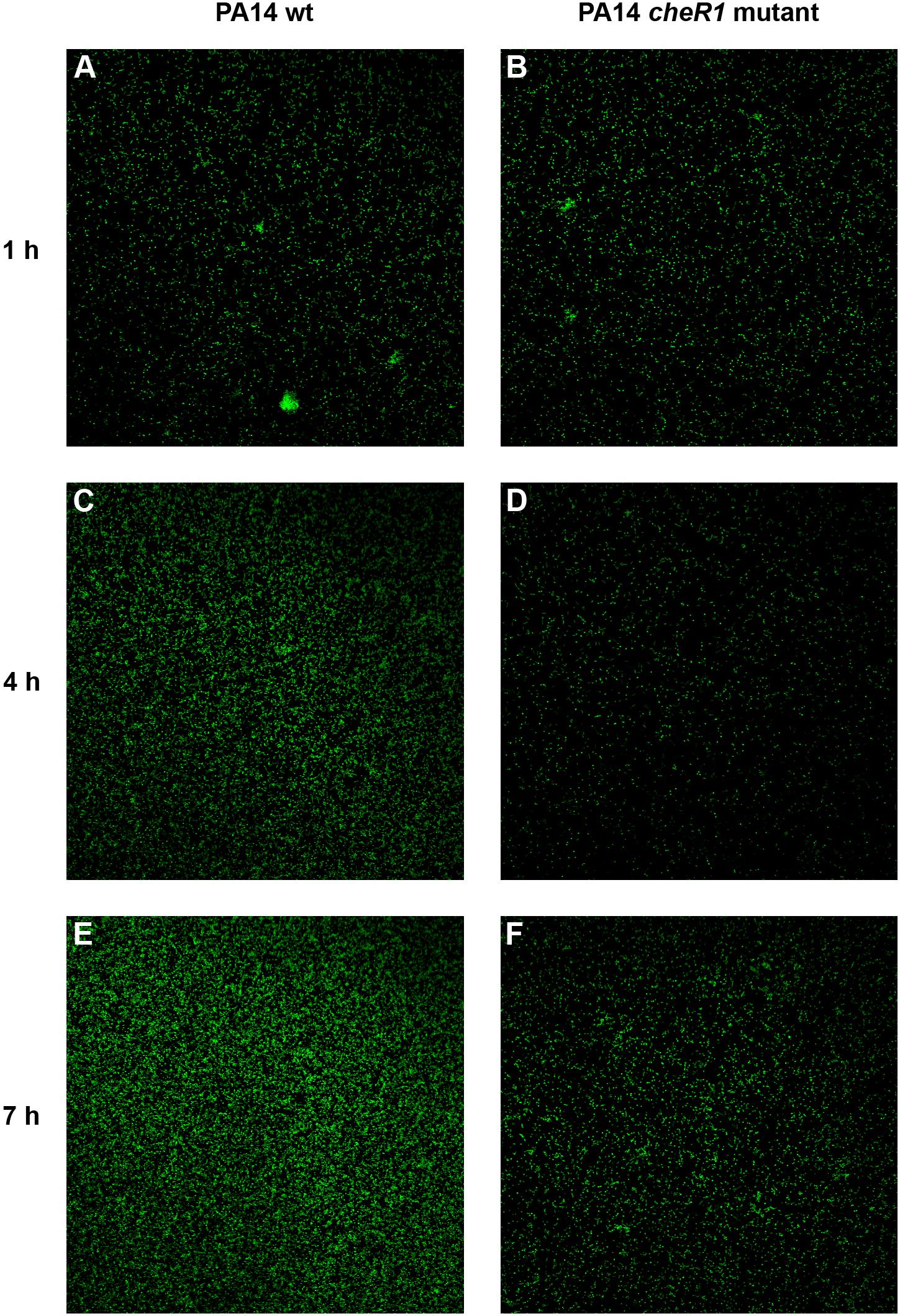

Supplement: Figure S1 — Substratum coverage by GFP-tagged bacteria as monitored by CLSM. The coverage of the well-bottom of a 96-well plate was monitored after (A, B) 1 h, (C, D) 4 h and (E, F) 7 h of growth in LB at 37°C. The cell clusters observed in (A) and (B) are likely to originate from cell clumps of over night grown pre-cultures used for inoculation. (A, C, E) PA14 wild-type and (B, D, F) PA14 cheR1 transposon mutant. (TIF) [file pone.0018184.s001.tif]
